# Does presence of R wave forces on surface electrocardiogram predict myocardial viability in patients with coronary disease and left ventricular dysfunction? Correlation with cardiac MRI

**DOI:** 10.1186/1532-429X-15-S1-E62

**Published:** 2013-01-30

**Authors:** Gurjit Singh, Sabha Bhatti, Karthikeyan Ananthasubramaniam

**Affiliations:** 1 Cardiovascular Medicine, Henry Ford Hospital, Detroit, MI, USA

## Background

Clinicians use presence of Q waves as a sign of transmural infarction although this is an imperfect marker to predict lack of myocardial viability (MV). We hypothesized that presence of R waves as a marker of electrical activity on surface electrocardiogram (ECG) may be a sensitive marker for presence of MV as determined by delayed enhanced cardiac magnetic resonance (DE-CMR) imaging.

## Methods

Retrospective study of 105 patients referred for assessment of MV by CMR with prior diagnosis of CAD and LV dysfunction. Cine steady state-free precession (SSFP) sequences and DE-CMR images were performed using standard protocols. Baseline ECG's were analyzed blinded to DE-CMR in 2 ways: presence of any R waves in ≥ 2 contiguous anterior (V1-V5; LAD), inferior (II, III, aVF; RCA) and lateral leads (I, aVL, V6; LCX) and ≥ 3 contiguous lead in a similar fashion. ECG's were also analyzed for presence of Q waves and QR complexes in a similar fashion. DE was quantified using a standard 17-segment model. Anterior wall was considered viable when > 3 out of 7 segments had <50 % DE in each segment and > 2 out of 5 segments had < 50 % DE in the inferior and lateral walls.

## Results

Mean age of the cohort was 63.2 ± 11.6 years and mean left ventricular ejection fraction was 33.5 ±13.6 %. With our viability definition, 83.8 % patients had viability in the anterior and inferior segments along with 95.2 % in the lateral segments. Sensitivity of any R wave to predict viability in the anterior wall was 96.5 % with a positive predictive value of 85.0 % but a poor specificity of 13.3%. Sensitivity of R waves for the inferior and lateral wall was 94.3 % and 100.0 % each. There was no substantial difference in sensitivity or PPV using both methods of R wave presence. (Table 1) QR complexes in patients with anterior Q waves worsened the sensitivity to 48.0 % with a specificity of 33.3 % to predict MV in LAD territory as shown in Table 1.

**Table 1:** Sensitivity and specificity of R/QR waves to predict myocardial viability

R waves	Sensitivity (%)	Specificity (%)	PPV (%)	NPV (%)
Anterior Any R (≥ 2 leads)	96.5	11.7	85.0	40.0

Any R (≥ 3 leads)	89.7	17.6	84.9	25.0

Inferior Any R (≥ 2 leads)	94.3	0.0	83.0	0.0

Any R (≥ 3 leads)	82.9	5.8	82.0	6.2

Lateral Any R (≥ 2 leads)	100.0	0.0	95.2	0.0

Any R (≥ 3 leads)	95.0	20.0	95.9	16.6

QR waves	SENSITIVITY (%)	SPECIFICITY (%)	PPV (%)	NPV (%)

Anterior Any R (≥ 2 leads)	48.0	33.3	66.6	18.7

Inferior Any R (≥ 2 leads)	80.0	0.0	66.6	33.3

Lateral Any R (≥ 2 leads)	85.7	0.0	85.7	0.0

## Conclusions

Presence of R waves on surface 12 lead ECG in patients with ischemic cardiomyopathy is a highly sensitive and simple tool to predict MV in all 3 major coronary artery territories with excellent positive predictive value. Clinicians could use this simple ECG tool to potentially channelize patients to further confirmatory viability testing when decision for revascularization based on left ventircular dysfunction is unclear.

## Funding

None

